# Editorial: The kidney in auto-immune and auto-inflammatory processes: Definitions, mechanisms, and biomarkers

**DOI:** 10.3389/fmed.2022.1129021

**Published:** 2023-01-10

**Authors:** Augusto Vaglio, Marco Gattorno, Stephen McAdoo, Laura Piera Obici, Gian Marco Ghiggeri

**Affiliations:** ^1^Nephrology and Dialysis Unit, Meyer Children's Hospital, Florence, Italy; ^2^Department of Biomedical Experimental and Clinical Sciences, University of Firenze, Florence, Italy; ^3^Center of Autoinflammatory Diseases and Immunodeficiencies, Department of Pediatrics and Rheumatology, IRCCS Istituto G. Gaslini, Genoa, Italy; ^4^Centre for Inflammatory Disease, Department of Immunology and Inflammation, Imperial College London, Hammersmith Campus, London, United Kingdom; ^5^Imperial College Renal and Transplant Centre, Imperial College Healthcare NHS Trust, Hammersmith Hospital, London, United Kingdom; ^6^Amyloidosis Research and Treatment Centre, IRCCS Fondazione Policlinico San Matteo, Pavia, Italy; ^7^Division of Nephrology, Dialysis, Transplantation, IRCCS Istituto Giannina Gaslini, Genoa, Italy

**Keywords:** autoimmune renal diseases, autoinflammatory diseases, lupus nephritis, ANCA associated vasculitis, membranous nephropathy, IgA vasculitis, amyloidosis

Autoimmune and autoinflammatory processes represent the starting mechanism of renal damage in a number of pathologies of the kidney. Autoimmune processes include diseases in which autoantibodies represent the major pathogenic mediator of lesions that, in general, involve glomeruli; auto-inflammatory conditions are, instead, characterized by the activation of an inflammatory cascade diffuse to all the renal compartments that may occur as a manifestation of genetic background or, more frequently, as the result of an inflammatory trigger.

There are some overlaps between autoimmunity and inflammation in the mechanisms that lead to renal damage: a clear distinction between the two processes does not exist and it makes sense to attempt a classification only for clinical purposes. Systemic Lupus erythematosus (SLE) with lupus nephritis (LN), anti-neutrophil cytoplasmic antibody (ANCA)-associated renal vasculitis and membranous nephropathy (MN) are representative of the autoimmune group. Inflammatory and auto-inflammatory diseases that can involve the kidney include rarer conditions such as IgG4 related pathologies, PFAPA, and hemophagocytic syndromes, which are characterized by a diffuse process in which the kidney is one of the target organs but other manifestations may occur. IgA vasculitis (formerly Henoch-Schönlein purpura) with glomerulonephritis is a condition with still unclear pathogenesis that is characterized by multisite localizations of the vasculitic process (with deposition of IgA mainly in skin and kidneys) and active inflammatory elements present at both sites (Figure 1).

**Figure 1:**
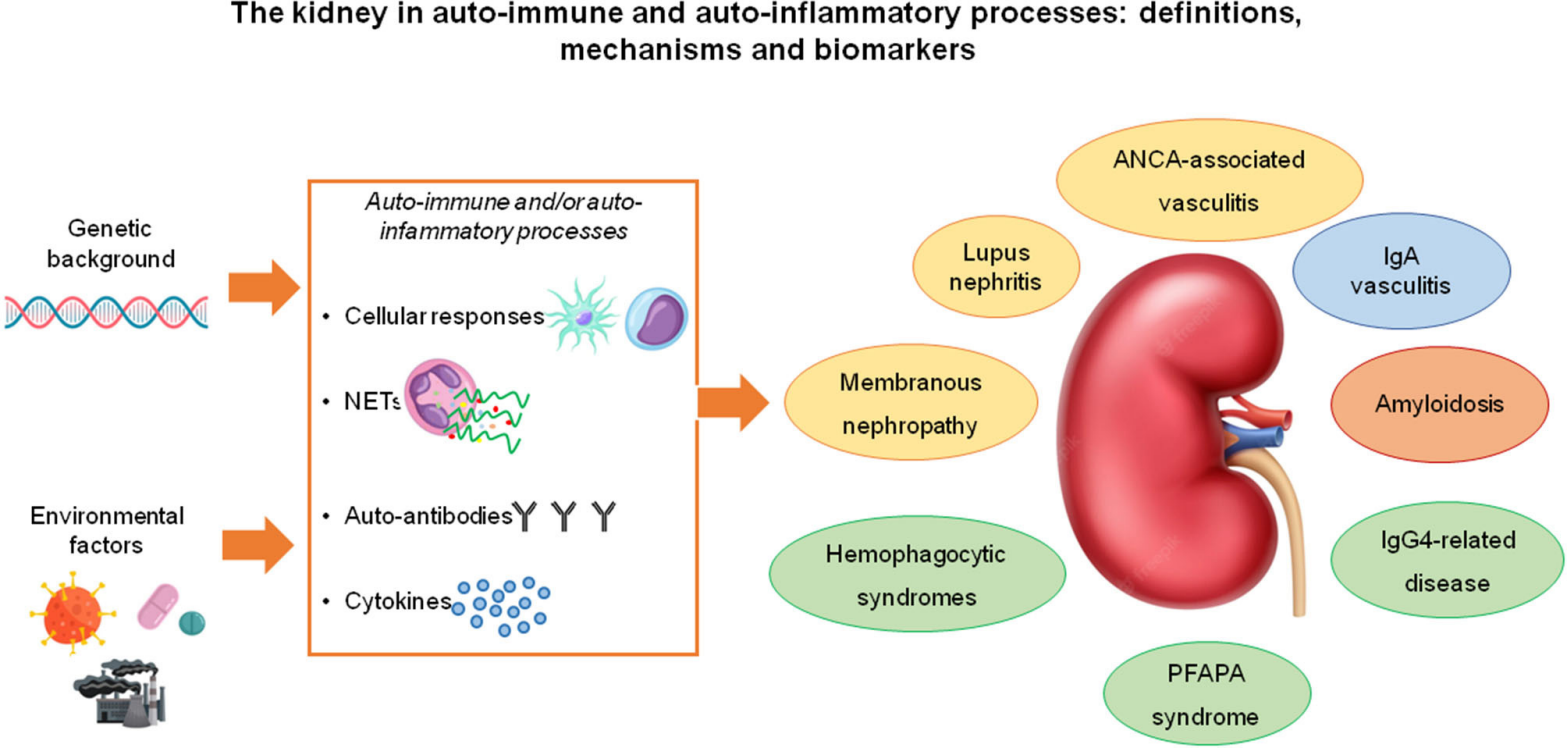
Most of the immune-mediated, systemic diseases that affect the kidney are “complex diseases” that result from the interaction between genetic susceptibility factors and environmental factors. The pathogenic mechanisms through which they cause kidney disease involve T-cell and macrophage/dendritic cell-mediated immunity, neutrophil activation and NET (neutrophil extracellular trap) formation with the exposure of auto-antigens and the consequent stimulation of autoimmune responses, the production of autoantibodies and the secretion of immuno-modulatory cytokines. The diseases included in this spectrum may have well-known pathogenic autoantibodies (e.g., lupus nephritis, ANCA-associated vasculitis, membranous nephropathy- all highlighted in yellow), pathogenic immune-complexes (IgA nephropathy, highlighted in blue), pathogenic monoclonal or polyclonal proteins able to form amyloid (amyloidosis, in orange), autoinflammatory or yet unknown pathogenic hallmarks (all the conditions highlighted in gray).

This Research Topic of Frontiers has the ambitious aim to cover at least some of the above issues including either pathogenetic mechanisms and clinical aspects. The selection of articles includes (1) a larger section addressing classical autoimmune diseases (SLE/LN and ANCA associated vasculitis) with the proposal of new classification criteria for Eosinophilic Granulomatosis with Polyangiitis (EGPA), a condition between ANCA-associated vasculitis and hypereosinophilic diseases. In terms of autoimmunity, a few papers describe the cells and regulatory molecules involved in the pathogenesis of renal damage, and a special section has been devoted to neutrophil extracellular traps (NETs), a specific mechanism involved in the formation of anti-DNA and other auto-antibodies. (2) The collection includes an article focussing on the involvement of complement and bilirubin in the progression of IgA vasculitis. (3) An overview of auto-inflammatory (PFAPA) and hemophagocytic syndromes. (4) A paper devoted to IgG4 related renal disease and, finally (5) a study on renal amyloidosis.

## 1. Renal autoimmune conditions

### 1.1. Lupus nephritis

Lupus nephritis is the most typical autoimmune glomerulonephritis. It is determined by intra-glomerular deposition of antibodies and complement that bind the basement membrane and modify *per se* permeability of the glomerular barrier causing proteinuria and activating an inflammatory cascade leading to the proliferation of extra and intra-glomerular cells and the development of sclerosis. Studies on the cells and cytokines involved in the inflammatory phase of LN focused on the Th17/IL17 axis as a specific mediator associated with CD8^+^ T cell hyperactivation. These aspects have been reviewed by Paquissi and Abensur who reported high levels (and high percentages) of IL-17 expressing cells in both the circulation and glomeruli of patients with LN that correlated with general parameters of SLE activity such as the SLEDAI index and with the specific renal parameters of renal damage such as GBM thickening. IL-17 can also be considered the determinant of a complex series of events within all the compartments of the kidney including modulation of the cytoskeleton in glomerular cells, activation of oxidative stress, and stimulation of the synthesis of other inflammatory cytokines such as IL-23 (1). All these effects can amplify the mechanisms of glomerular damage typical of LN (2). As correctly recognized by the authors, several association studies provide indirect support on the implication of the Th17/IL-17 axis in LN but not direct evidence. Assessing the efficacy of new drugs that play a specific inhibitory effect on IL-17A could provide an answer to this question. The first inhibitor, secukinumab, is currently being tested for safety and efficacy in a trial on the cutaneous manifestation of SLE (NCT03866317) that is preliminary to assessing its effects on other organs (3).

Wiechmann et al. investigated the amount of CD8^+^ T cells in LN and their activation status as reflected by the expression of CD107a. Positivity for CD107a in CD8+ T cells is considered a marker of killing activity given by the capability of CD107a positive cells to secrete perforin and granzyme B (4). In analogy, high circulating CD107a+ CD8^+^T-cells in patients with SLE correlated with the SLEDAI activity index. In the kidney of LN patients, CD107a+ CD8^+^T-cells were localized almost completely in tubule-interstitial areas and correlated with proteinuria and the chronicity index. These data lead us to consider CD107a+CD^+^8 T cells as a major effector of cytotoxic activity in the tubule interstitium, which represents a very important functional compartment of the kidney. Targeting CD107a could be a possible evolution of new therapies.

Two papers in the Research Topic were dedicated to the mechanisms leading to the formation of autoantibodies; one specifically addressed the reasons for the IgG2 isotype switch that is typical of LN. It is well known that autoantibodies are involved in the pathogenesis of LN target, in the majority of cases, dsDNA and other intracellular proteins. In both cases, activation of the autoimmune process requires that target antigens are exposed to the microenvironment. The mechanisms for their exposition are poorly understood. NETosis is a process characterized by extracellular exposure of dsDNA from the intracellular compartment of neutrophils and is, therefore, of interest for anti-dsDNA formation. Neutrophil extracellular traps (NETs) contain, besides dsDNA, many other intracellular proteins that become potential antigens for autoimmunity in LN. In their contribution to this collection, Bruschi et al. review many aspects related to the composition and metabolism of NETs in healthy and pathological conditions. In normal conditions, the formation of NETs represents a physical barrier made of DNA, nucleosome and other intra-neutrophil proteins that is functional to protect from external infectious organisms (suicidal NETs) (5). In this context, NETosis is considered an early step of innate immunity. Another pathway for NET generation in SLE is by immunologic triggers (sterile NETs) in which case NETs are directly connected with the overall autoimmune activity. Once formed, NETs are removed by DNase1 and 13 that digest DNA and it is the imbalance between production and removal of NETs (for DNase deficit) that in SLE leads to the abnormal production of anti-DNA antibodies. *DNase13* mutations have been associated with familial LN and, more generally, the circulating inhibitors of DNases (probably antibodies) have been detected in patients with SLE in association with defective removal of DNA (6). A significant aspect of NETs is that they contain, besides DNA, other intracellular proteins, such as enolase and annexin A1, that are targets of autoantibodies whose levels are high in the circulation and micro-dissected glomeruli of patients with LN (7, 8). Bertelli et al. reported new experiments that aimed to explain why the majority of autoantibodies in SLE are of the IgG2 isotype that requires a class switch recombination step. The basic hypothesis made by the authors was that this passage implicated NETs. They show that NETs purified from SLE patients induced an important release of soluble IgG2 by the naïve B cells of SLE patients that did not take place in healthy donors, implying the existence of regulatory factors linked with SLE. In parallel, NETs stimulated *ex vivo* IgG2 isotype class switch through the induction of T-bet, a transcriptional factor that was expressed by an “atypical memory” CD19 clone highly expanded in patients with SLE. T-bet acts in association with TLR-9, with other stimulating factors such as type I IFN, and with components of the MyD88-related pathways. T-bet activity was also associated with other antagonizing factors. These data strengthen the notion that NETs are implicated in the autoimmune milieu, which characterizes LN, and indicate that the regulatory mechanisms of the IgG2 isotype class switch could become a target of specific therapies.

A unique manuscript in this issue addresses the clinical aspects of SLE. Bao et al. describe a rare association between SLE/LN and mantle cell lymphoma, which is an aggressive B-cell non-Hodgkin lymphoma that is usually unresponsive to common therapies. The association of mantle cell lymphoma with glomerular diseases (i.e., minimal change disease, membrano-proliferative, and ANCA associated glomerulonephritis) has already been reported (9) but this is the second case of an association with LN that could suggest a pathogenic link with B-cell anomalies. On a more clinical vein, it is of interest that renal symptoms are remitted after the first chemotherapy, in concomitance with the remittance of the lymphoma itself.

### 1.2. Membranous nephropathy

Membranous nephropathy is a primary autoimmune disease limited to the kidney and characterized by sub-epithelial deposits of autoantibodies and complement. In this view, MN shares some pathologic features with Class V LN such as the same sub-epithelial deposition of autoantibodies. MN causes nephrotic syndrome and represents a main leading factor for the evolution of chronic renal failure. In the last decade, several target antigens of the auto-antibodies responsible for MN have been characterized which represents a significant evolution in the pathogenesis of the disease. Anti-PLA2R1 is the most common (present in 65% of patients), followed by anti-TSHD7A (3%) and by several others which have a minor numerical impact being, in some cases, described in less than 5 patients (10, 11). Most of the antigens above are proteins expressed in the glomerular membrane, which explains why their deposition causes proteinuria. Antibodies targeting intracellular antigens such as anti-SOD2 have been reported and considered an adjunctive reason for the evolution of tissue lesions, given the well-known anti-oxidant power of this molecule (12). In their study, Hu et al. describe particular aspects of the reparative process related to the expression of macrophage sub-populations in renal tissue of a numerically significant cohort of 55 patients with MN. They focused on M2a, M2B, and M2c macrophages which are the major cells involved in the anti-inflammatory and reparative processes in glomeruli and tubule-interstitial areas. Correlations between a number of macrophages in each category and pathologic parameters (IgG1 and C3 staining) were reported and interpreted as proof of the involvement of these cells in post-acute damage. This observation re-opens a topic that attracted the interest of pathologists a few years ago when some studies proposed that the degree of tubulointerstitial macrophage infiltration determines the prognosis of MN (13, 14).

### 1.3. ANCA-associated renal vasculitis

ANCA-associated renal vasculitis is less common but usually more severe than LN and four papers in this Research Topic are dedicated to its pathogenetic and clinical aspects. Wang et al. evaluate the potential association with Annexin A1 in 66 patients with AAV, 31 in the active state of the disease, and 35 in remission. The intent was to characterize the activation state of neutrophils since Annexin A1 is a circulating protein that has a main role in the regulation of neutrophil trafficking, adhesion, and transmigration (15) and contributes, in this way, to the resolution of inflammation (16). In this study, expression of Annexin A1 was found, besides in neutrophils, also in monocytes/macrophages and T cells, as well as in several renal cell types, i.e., podocytes, mesangial cells, and tubular epithelia, widening the significance of this protein in renal pathology. Plasma levels were high in active AAV and correlated with the proliferation of intrinsic glomerular cells (podocytes, mesangial cells) and circulating neutrophils and macrophages. The conclusions were that renal and cell Annexin A1 is stimulated by inflammation and potentially participates in its resolution based on the strong anti-inflammatory effect of this protein. The unique caveat of the study was the concomitance of steroid therapies since it is well known that Annexin A1 levels in circulating neutrophils are under the control of glucocorticoids (endogenous and exogenous) that involve the Annexin A1 receptor (ALXR), the glucocorticoid-induced leucine zipper gene (*GILZ*) (17) and probably other cytokines such as IL6 (18).

Xia et al. evaluated the accuracy of a score for predicting the evolution of ANCA-associated renal vasculitis into end-stage renal disease, also known as the ANCA Renal Risk Score (ARRS). They utilized a meta-analysis approach and found 1.568 ANCA positive patients with glomerulonephritis reported in 11 studies that utilized the ARRS for predicting the clinical outcome. Pooled ANCA-GN patients were subdivided into three different groups with low (score 0–1), medium (score 2–7), and high ARRS (score 8–11), and they found a cumulative number of patients with end-stage renal disease after 60 months of follow up of 5%, 22%, and 59% respectively. The pooled sensitivity of the test in predicting ESRD was 98% with a specificity of 30% for ARRS > 2, and 58% with a specificity of 86% for ARRS > 8. This led to the conclusion that the ARRS performs well in predicting poor outcomes in ANCA-GN patients.

Xu et al. described a 14-year-old girl with microscopic polyangiitis who developed intracerebral hemorrhage associated with posterior reversible encephalopathy syndrome (PRES). This girl presented abrupt seizures in concomitance with severe hypertension and underwent remission after combined therapy with methyl-prednisolone pulses, plasmapheresis, anti-seizure, and anti-hypertensive medications. The authors reviewed the literature and found 6 cases that presented the same association. The importance of this finding is that in the presence of microscopic polyangiitis, cerebrovascular complications may occur and must be correctly recognized since they can revert after appropriate therapies.

### 1.4. Eosinophilic granulomatosis with polyangiitis

Eosinophilic granulomatosis with polyangiitis (EGPA) is a rare multisystem disease that is considered to be between an inflammatory disorder with hyper-eosinophilia and a classical ANCA-associated vasculitis. Fagni et al. attempted a classification of EGPA based on ANCA positivity (mainly MPO) in patients with clear vasculitic manifestations vs. patients with generalized symptoms more linked with eosinophilia and ANCA-negativity. Renal involvement was also predominant in the former group. Clinically, EGPA is characterized by classical signs of eosinophil activation without inter-group difference where cardiac, neural, pulmonary, and vascular manifestations correlated with the entity of eosinophil infiltration (cardiac is the most frequent cause of death) (19). Genome-wide association studies supported the view that there is a dualism between being ANCA positive and having negative EGPA, showing an association with HLA class II variants in the former group (20), suggesting a link with CD4^+^ T-lymphocyte activation. The predominance of IL17 and Th17 cells in ANCA positive patients would create an amplification loop promoting neutrophil tissue recruitment and activation that is the basis for anti-MPO formation (21). By contrast, ANCA negative EGPA was associated with the *IRF1/IL5* that interacts with IL-4 and IL-5 promoter regions and is directly linked with eosinophil activation and asthma (20, 22). Since a clear phenotypic differentiation is not the rule in EGPA patients, the authors concluded that the application of a strict dualism cannot yet be translated into routine clinical practice and remains a goal for future studies.

## 2. IgA vasculitis with glomerulonephritis

Cutaneous IgA vasculitis is a common disease in children, characterized by the formation of typical purpuric lesions of the skin that usually resolve after a few weeks. In adolescents and adults, cutaneous IgA vasculitis, although rare, is frequently associated with renal lesions initially limited to glomerular proliferation but may evolve to glomerulosclerosis and tubule-interstitial with worsening renal function. IgAV-glomerulonephritis (GN) is characterized by urinary abnormalities such as hematuria (macro and micro) and variable degrees of proteinuria (23). Clinical or laboratory features compatible with IgAV-GN mandate kidney biopsy for the confirmation and staging of tissue lesions.

Two papers address the key problem of the severity of IgAV-GN in adults and underline the need to develop predictors of clinical outcomes. Romero et al. determined the amount of C4d deposition in glomeruli of 120 adults with IgAV-Gn and found that it was correlated with either the index of renal disease activity or clinical long term outcome. C4d is generically considered an index of poor prognosis in patients with isolated IgA nephropathy (24) and it seemed important to confirm the importance of this parameter in adults with IgAV-GN. C4d is a complement fraction with no clear biological function that derives from the degradation of complement within the lectin pathway. The concomitant presence in the glomeruli of C4d and mannose modified IgA has pathogenetic significance for the activation of this alternative complement cascade (25). Glomerular C4d was found in 23% of patients with IgAV-GN in association with increased mesangial proliferation and baseline proteinuria, which were considered a predictor of poor prognosis and justified a more aggressive therapeutic approach. The renal outcome in terms of proteinuria and renal function in the two cohorts of C4d+ and C4d- patients evaluated retrospectively was comparable, limiting the interest on C4d (and more in general on compliment) as a biomarker of the progression of IgAV-Gn. The second paper published in the Research Topic by Tan et al. proposed bilirubin as a protective factor for the progression of IgAV-Gn based on the antioxidant, anti-inflammatory, and vascular protective functions of this molecule. Previous reports have already demonstrated that low bilirubin levels are associated with poor evolution of IgA GN and diabetic nephropathy and are, more in general, correlated with the progression of CKD (26, 27). The authors studied 189 young and young adults with IgAV-GN (age >16 years) and found, with multivariate Cox analysis, that the serum bilirubin was an independent protective factor for renal survival where the composite endpoint was defined as a 50% decline in e-GFR, end-stage renal disease or death. The result was confirmed in 89 patients who were matched for baseline clinicopathological manifestations and treatments except for serum bilirubin. In this case, the retrospective model of the study also refrains from definite conclusions still pointing to unexpected functions of this common molecule. The protective activity of bilirubin can be explained by the known inhibitory effect that this molecule plays on complement and on cytokines.

## 3. Auto-inflammatory and hemophagocytic syndromes

Two papers in this section address the main issue of renal involvement in two inflammatory conditions, one with a genetic origin, i.e., Heterozygous mutations of the proline-serine-threonine phosphatase interacting protein 1 gene (*PSTPIP1*) and the other, i.e., hemophagocytic syndrome (HPS) or hemophagocytic lymphohistiocytosis.

Borgia et al. described a 22-year-old male presenting the genetic imprinting of a variant of the PSTPIP1 syndrome also known as *PSTPIP1*-associated myeloid-related proteinemia inflammatory syndrome or PAMI (p.E250K and p.E257K) (28, 29). Renal involvement is part of the clinical picture that may vary from vasculitis to more generic tubulo-interstitial involvement. In line with a few reports in the literature on renal involvement in PAMI, the young adult here described developed glomerular lesions compatible with Focal Segmental Glomerulosclerosis (FSGS) and consequent renal failure (30) and was treated with the association of Canakinumab and Tacrolimus. The effects of this association were particularly satisfactory on the general inflammatory status and on skin lesions, which reverted significantly, whereas they were only partially effective in treating proteinuria and renal function that only stabilized. The pathogenesis of FSGS is unclear (31), the participation of the IL 1 pathway has been proposed and it is indirectly supported by the positive effects produced by blockers of the IL-1β/IL-1R1 signaling pathway (32). Therefore, this case report strengthened this pathogenetic concept.

HPS is a rare syndrome characterized by fever, pancytopenia, hepatosplenomegaly, liver dysfunction, and by the presence of nonmalignant macrophages infiltrating the bone marrow. A generic renal involvement is frequently reported without a clear site-specific pathologic definition (33). Roccatello et al. attempted to widen the spectrum of HPS to renal-limited forms, describing four cases with severe renal involvement, two presenting with AKI and the other two with chronic renal failure of varying degrees, telescopic urinary sediment, with hematuria and proteinuria and the presence of CD68^+^ infiltrating cells in the renal tissue as common features. Hyperferritinemia and platelet consumption were present in only one case. The renal aspects of this series of patients are in line with what is reported in wider literature on renal involvement in HPS (60% of patients presented with AKI and 40% with the nephritic syndrome) (34, 35). Other renal histology reports also indicate that inflammatory or ischemic tubule-interstitial lesions, in general, correlate with AKI, and podocytopathies with collapsing lesions and/or thrombotic microangiopathy in some patients with a chronic outcome of renal failure; macrophage infiltration, as here reported, has been only occasionally described (35). The definition of “renal limited HSP” requires confirmation in other cohorts of patients before being consolidated as a particular aspect of HSP.

## 4. IgG4 related disease

IgG4-related disease (IgG4-RD) is a rare inflammatory condition (1:100.000) characterized by lymphoplasmacytic infiltrate in many organs, obliterative phlebitis, and storiform fibrosis; eosinophilia and increased levels of IgG4 are also common (36, 37). Responsiveness to steroids is a major clinical characteristic of IgG4-RD (38).

Capecchi et al. described 4 cases of IgG4-RD with prevalent renal involvement that summarize the expression this syndrome may have in the kidney. Focal tubulointerstitial multifocal lymphocytic infiltrates, where IgG4 + plasma cells predominated, and tubulitis were hallmarks of the disease. Typical storiform or “cartwheel fibrosis” represents the evolution of tubulointerstitial lesions to more chronic and irreversible alterations that preceded end stage renal failure (39). Membranous glomerular lesions usually coexist with tubulointerstitial nephritis and nephrotic proteinuria. Since primary membranous nephropathy is also typically characterized by IgG4 sub-epithelial deposits the differential diagnosis between the two conditions may pose some difficulties and is merely based on the presence of TIN and the association with other organ involvement that is always absent in primary membranous nephropathy. Retroperitoneal fibrosis evolves into obstructive nephropathy that may occur in IgG4-RD, an additional possible cause of renal failure (39). In general, this condition is clinically silent and causes progressive chronic renal lesions. Acute renal obstruction is in general characterized by severe pain and may be easily detected by renal ultrasound, and ureteral stenting or percutaneous nephrostomy allows rapid resolution of the obstructive state.

## 5. Amyloidosis

Secondary amyloidosis (AA amyloidosis) is a common complication of autoinflammatory diseases as well as of organ and site-specific chronic conditions that occur frequently such as those involving the bowel (IBD) and joints (rheumatoid arthritis, ankylosing spondylitis)(40). Serum Amyloid A (SAA) is an acute phase protein, produced by the liver and macrophages in response to an inflammation under the transcriptional control of pro-inflammatory cytokines, particularly tumor necrosis factor (TNF) alpha, interleukin-1 (IL-1) beta and IL-6 (41, 42). SAA circulates and deposits in many organs where it forms misfolded aggregates of proteins and fibrils that are resistant to degradation. The heart, kidney, liver, gastrointestinal tract, and peripheral nerves are targets of this process, which is chronic, and sustained by the failure of the degrading systems to remove amyloid. Early therapeutic interventions with TNF-blockers and/or anti-interleukin agents seem the most promising approaches, aiming to block SAA formation before deposition (42).

Another article in this issue by Eriksson et al. describes the encouraging effects of the anti-IL6 receptor antibody tocilizumab in two patients with long-standing ankylosing spondylitis who developed proteinuria and presented renal deposits of amyloid in vessels and glomeruli. At the start of the therapy, the inflammatory parameters had a rapid drop, with normalization of SAA levels that were followed by an improvement of either proteinuria and renal function after 18 and 54 months follow up. While tocilizumab had been already utilized in AA secondary to rheumatoid arthritis (43), these two patients were the first with ankylosing spondylitis-related AA amyloidosis to be treated with this agent and the positive effect on renal parameters supports a more diffuse use of this drug in AA secondary to ankylosing spondylitis. Some pathogenetic clues are outlined by these data, indicating that a late intervention to block SAA production may activate a beneficial reparative mechanism in the kidney. In other words, it seems that a divergent association between amyloid accumulation in the kidney, which is irreversible, and renal symptoms (i.e., proteinuria, renal function) exists, suggesting the acute toxic effect of SAA in the kidney (44, 45). The progressive nature of renal amyloidosis may be blocked at a stage in which the function of the organ is still normal.

## Author contributions

All authors listed have made a substantial, direct, and intellectual contribution to the work and approved it for publication.
